# Bullous Pilomatricoma of the Neck With Lymphatic Involvement: A Case Report

**DOI:** 10.7759/cureus.104260

**Published:** 2026-02-25

**Authors:** Guillermo Roa Álvarez, Mario Shuchleib Cukiert, Felix R Hernández Altamirano, Maria E Vega Memije

**Affiliations:** 1 School of Medicine and Medical Sciences, Tecnologico de Monterrey, Mexico City, MEX; 2 Department of Dermatology, Hospital General “Dr. Manuel Gea Gonzalez”, Mexico City, MEX; 3 Department of Dermatopathology, Hospital General “Dr. Manuel Gea Gonzalez”, Mexico City, MEX

**Keywords:** bullous pilomatricoma, hair follicle matrix tumor, lymphangiectasia, lymphatic abnormalities, lymph node involvement, pilomatricoma, skin neoplasms

## Abstract

Pilomatricoma is a benign adnexal neoplasm derived from hair matrix cells, most frequently occurring in children and young adults. Although it typically presents as a firm, well-circumscribed dermal nodule, several uncommon clinical variants have been described. Among these, the bullous (lymphangiectatic) variant is particularly rare and may clinically mimic cystic or vascular lesions, creating diagnostic difficulty. We report the case of a 19-year-old male who presented with a rapidly enlarging, translucent, bullous lesion in the posterior cervical region. Physical examination revealed a biphasic lesion consisting of a soft, fluctuant superficial component overlying a firm, mobile nodule, with positive transillumination. Ultrasonography demonstrated a well-defined subcutaneous mass measuring approximately 2.0 cm in depth, with mixed echogenicity and internal calcifications, without evidence of vascular flow. Complete surgical excision was performed. Histopathologic examination confirmed the diagnosis of bullous pilomatricoma, showing characteristic basaloid cells transitioning into ghost cells, dystrophic calcification, marked dermal lymphangiectasia, and foreign-body granulomatous reaction. Notably, pilomatricoma debris associated with granulomatous inflammation was identified within adjacent lymph node tissue, representing an exceedingly rare and previously unreported finding. Surgical margins were free of tumor, and no recurrence was observed at six-month follow-up. This case broadens the recognized clinical and histopathologic spectrum of bullous pilomatricoma and highlights the importance of considering this entity in the differential diagnosis of cystic-appearing lesions of the head and neck. Complete surgical excision remains curative and is associated with an excellent prognosis.

## Introduction

Pilomatricoma, also known as pilomatrixoma or calcifying epithelioma of Malherbe, is a benign adnexal neoplasm derived from hair matrix cells. The lesion was initially distinguished from other adnexal tumors by Forbis and Helwig in 1961, redefining the original description by Malherbe and Chenantais and establishing it as a distinct entity within the group of hair follicle tumors [1]. Although pilomatricoma represents only about one percent of benign cutaneous lesions, it is one of the more common solid skin tumors in pediatric and adolescent populations, with nearly half of cases occurring before the age of 20 [2]. The head and neck region constitutes the predominant anatomic distribution, followed by the upper limbs, and lesions usually present as firm, well-circumscribed, slowly enlarging nodules that remain adherent to the overlying skin but mobile over deeper structures [3,4].

Despite its characteristic clinical behavior, pilomatricoma is frequently misdiagnosed before excision. Large retrospective studies have shown that fewer than one-third of lesions are correctly identified preoperatively, reflecting the wide range of clinical presentations and the overlap with other benign and malignant nodular conditions [5]. Definitive diagnosis, therefore, relies on histopathology, which typically demonstrates a biphasic composition of peripheral basaloid cells transitioning into central eosinophilic “ghost” or “shadow” cells, often accompanied by dystrophic calcification and foreign-body granulomatous inflammation [6].

Several clinical variants of pilomatricoma have been described over the years, including perforating, anetodermic, giant, and the bullous or lymphangiectatic subtype. The latter, first documented in 1943, accounts for approximately two percent of all pilomatricoma cases and is distinguished by a translucent, blister-like appearance that develops over the primary lesion [7]. The bullous phenotype reflects a combination of dermal lymphatic dilation, edema, and loss of elastic fibers secondary to mechanical obstruction or enzymatic degradation, producing a flaccid and often strikingly translucent superficial pouch that may mimic cystic hygroma or vascular anomalies [8,9].

Although the bullous variant is uncommon, its diagnostic implications are significant, as its unusual appearance may lead clinicians toward congenital, vascular, or cystic processes rather than an adnexal neoplasm. To date, descriptions of bullous pilomatricoma have emphasized dermal lymphangiectasia without deeper extension. The present case is notable for the novel finding of pilomatricoma debris within adjacent lymph node tissue, expanding the known clinical and pathological spectrum of this rare variant.

## Case presentation

**Figure 1:**
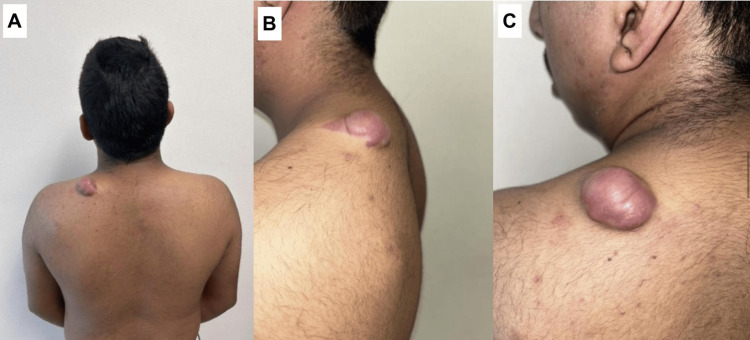
Clinical presentation of bullous pilomatricoma. A) Posterior view demonstrating a solitary, hemispherical, translucent-violaceous nodule located on the left posterior cervical/shoulder region. B) Lateral view highlighting the flaccid, bullous component overlying a deeper firm mass. C) Close-up oblique view showing the tense, dome-shaped, blister-like surface with underlying solid tumor.

**Figure 2:**
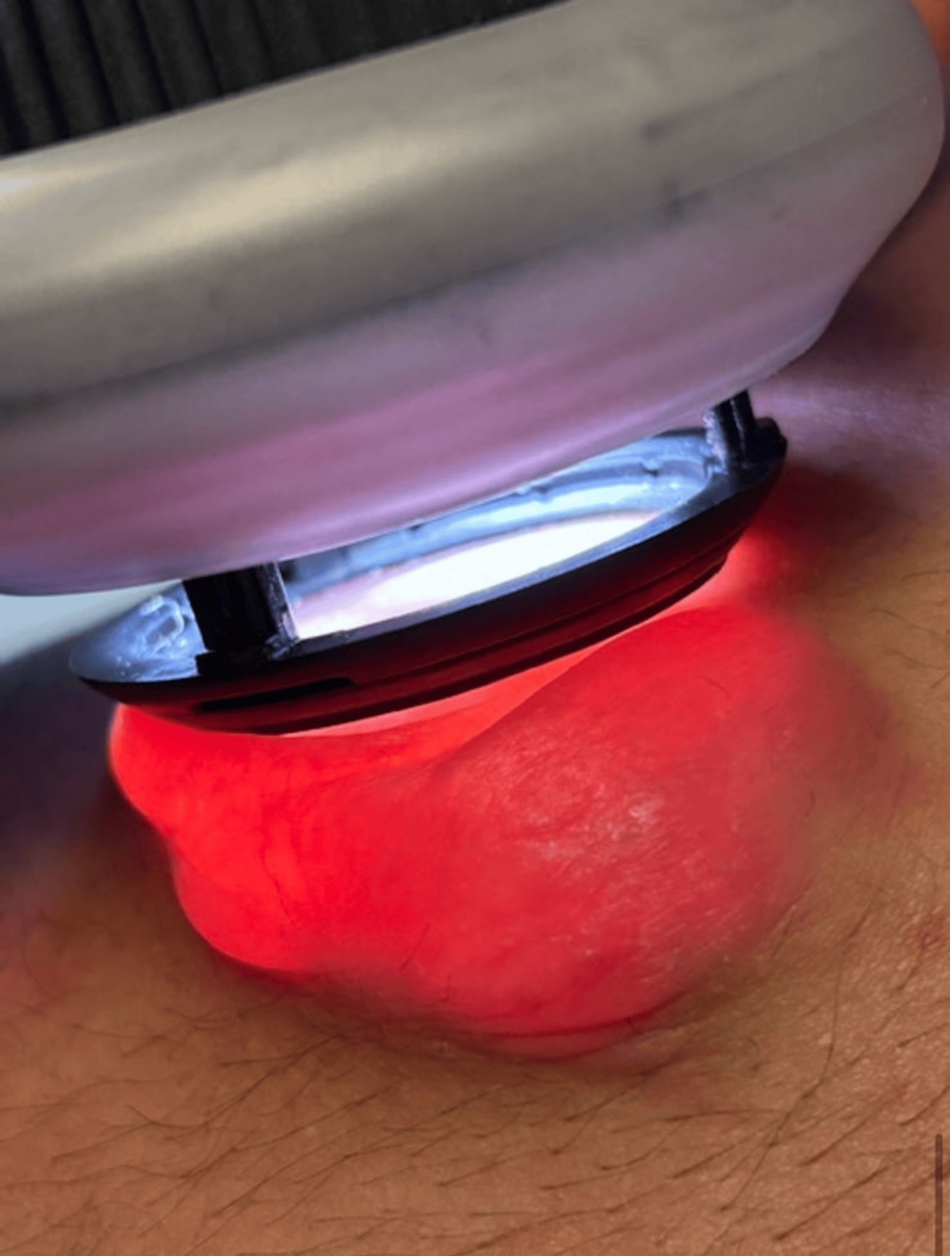
Positive transillumination test in the bullous pilomatricoma.

A 19-year-old male with no significant medical history presented with a nodular lesion on the left side of his neck (posterior cervical region) (Figure 1A, 1B). He first noticed the mass approximately two to three months prior, and it had gradually grown to measuring 2.3 × 1.5 × 2.0 cm at the time of presentation. It was otherwise asymptomatic, with no pain, and the patient denied any preceding trauma to the area. On physical examination, the lesion was an exophytic, hemispherical nodule measuring roughly 2.3 × 1.5 × 2.0 cm. The overlying skin was erythematous to violaceous and had a bullous, blister-like appearance (a flaccid sac over the nodule) (Figure 1C). Palpation revealed a striking biphasic consistency: the superficial component was soft, compressible, and fluctuant, whereas a deeper component was firm and mobile, not adherent to deeper tissues. Notably, a transillumination test was positive (Figure 2). No overlying skin ulceration or temperature change was present. The remainder of the neck exam was unremarkable, and no significant lymphadenopathy was palpated in the surrounding area. These imaging characteristics were diagnostically relevant, as the presence of internal calcifications and absence of Doppler flow favored pilomatricoma over vascular malformations or congenital cystic lesions.

Given the lesion’s cystic, translucent quality and underlying firmness, a provisional clinical diagnosis of bullous pilomatricoma was considered. The differential diagnosis for a cystic neck mass in this age group also included lymphatic malformations (cystic hygroma), hemangioma, epidermal inclusion cyst, and other benign skin tumors with cystic change. Dermoscopic examination did not reveal specific features in this case (no definitive calcific or vascular patterns). A high-resolution soft-tissue ultrasound was obtained, which showed a well-defined subcutaneous heterogeneous nodule approximately 2 cm in depth, with a hypoechoic superficial component and a hyperechoic solid center containing tiny calcific foci. No internal vascular flow was observed on color Doppler, and there was no extension into deeper structures of the neck. These sonographic findings supported the diagnosis of a localized encapsulated lesion rather than a vascular malformation or congenital cyst. With a benign tumor being likely, the decision was made to proceed with surgical excision for definitive diagnosis and treatment.

The patient underwent complete surgical excision of the lesion under local anesthesia. A fusiform ellipse of skin was removed, encompassing the nodule and a cuff of normal tissue. Intraoperatively, the superficial cystic portion was noted, and the lesion was removed in one piece without spillage of contents. The excised specimen was sent for histopathological analysis.

**Figure 3:**
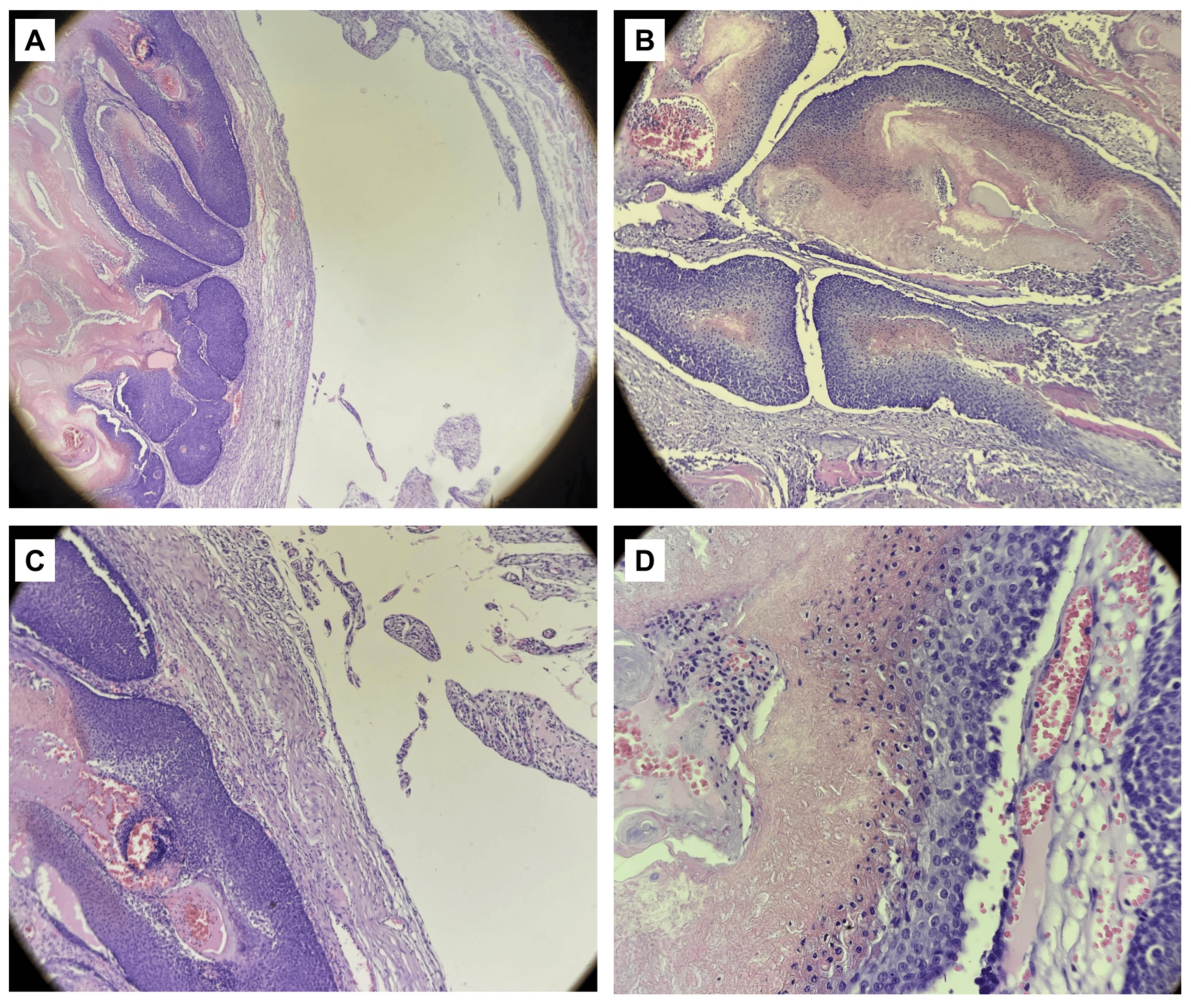
Histopathological features of bullous pilomatricoma (H&E). A) Low-power view (H&E slide in x40) showing a well-circumscribed, lobulated dermal tumor associated with marked superficial dermal edema and dilated lymphatic spaces. B) Intermediate magnification (H&E slide in x100) demonstrating epithelial tumor islands with peripheral basaloid cells and central eosinophilic ghost cells. C) (H&E slide in x100) Prominent lymphangiectasia and stromal edema surrounding the tumor nodules, correlating with the bullous clinical appearance.D) High-power view  (H&E slide in x400) showing ghost cells with abrupt keratinization and adjacent foreign-body granulomatous reaction.

Microscopic examination confirmed the diagnosis of pilomatricoma with bullous features. Sections showed skin with a cystic dilatation in the superficial dermis and an underlying well-circumscribed lobulated dermal tumor. The superficial dermis exhibited marked edema and widely dilated lymphatic vessels surrounding the tumor nodules, correlating with the bullous presentation. Beneath this, the tumor was composed of epithelial islands typical of pilomatricoma: nests of basaloid cells with dark, hyperchromatic nuclei at the periphery, gradually transitioning into pale eosinophilic shadow (“ghost”) cells that lacked nuclei (Figure 3A, 3B, 3C, 3D). Many of the ghost cells showed central areas of abrupt keratinization. Foci of dystrophic calcification were present within the tumor islands, and a considerable reactive inflammatory infiltrate was observed. Numerous multinucleated foreign-body giant cells and clusters of lymphocytes and histiocytes surrounded areas of keratin and calcified debris, indicating a granulomatous reaction to the tumor contents.

Notably, at the periphery of the lesion, there was a portion of residual lymphoid tissue consistent with a lymph node, in which similar granulomatous inflammation was seen, containing pilomatricoma debris. This finding suggested that the pilomatricoma had extended into or involved a regional lymphatic structure, a highly unusual occurrence for a benign pilomatricoma. No atypical cellular features, significant mitotic activity, or infiltrative growth pattern to suggest malignancy (pilomatrix carcinoma) were identified. The lesion was therefore diagnosed as a bullous (lymphangiectatic) pilomatricoma, ruptured with foreign-body granuloma formation. Surgical margins were clear of tumor cells. The post-operative course was uneventful, and the incision healed well. At a follow-up visit 6 months after excision, clinical examination revealed no evidence of recurrence; follow-up imaging was not deemed necessary given complete excision with clear margins and absence of clinical suspicion.

Timeline

The lesion was first noticed approximately three months prior to consultation, progressively enlarged over a two-to-three-month period, was surgically excised shortly after evaluation, and remained without recurrence at the six-month clinical follow-up.

## Discussion

Pilomatricoma is a benign adnexal neoplasm arising from hair matrix differentiation, and although its classical clinical and histopathologic features are well established, its bullous or lymphangiectatic variant remains a distinctly uncommon and diagnostically challenging presentation. Understanding the full pathobiology of this variant is essential to avoid confusion with congenital, vascular, or malignant processes. The current case not only displays the hallmark features of bullous pilomatricoma but also introduces an unprecedented finding of granulomatous lymph node involvement by pilomatricoma debris, broadening the known behavior of this tumor.

Classic pilomatricoma typically presents as a firm, well-circumscribed dermal or subcutaneous nodule composed of basaloid cells transitioning into eosinophilic, anucleate ghost cells, a biphasic pattern that reflects the aberrant keratinization pathway characteristic of hair matrix differentiation [6]. Over time, these lesions often demonstrate calcification and may incite a foreign-body giant cell reaction to keratin fragments [4]. Kaddu’s morphologic staging describes an evolutionary sequence from early basophilic phases through mixed basaloid-shadow cell stages to late calcified and ossified forms, capturing the dynamic biological processes underlying pilomatricoma maturation [10]. These stages are relevant to the bullous variant because partial rupture, keratin extrusion, and stromal remodeling are more likely to occur in later stages, potentially contributing to lymphatic changes.

The bullous or lymphangiectatic variant, while rare, is clinically significant because it diverges sharply from the firm, solid character of conventional pilomatricomas. In this variant, the overlying dermis becomes distended by lymphatic fluid, creating a translucent, flaccid sac that may resemble a blister. The pathogenesis of this bullous morphology appears multifactorial. Mechanical compression of superficial lymphatic channels by the underlying tumor may obstruct lymphatic flow, leading to marked lymphangiectasia, dermal edema, and fluid extravasation [8]. Additionally, tumor-associated or inflammatory MMPs may degrade collagen and elastic fibers, further weakening the dermal stroma and allowing lymphatic expansion [7]. Histologic examination of bullous lesions consistently reveals dilated lymphatic vessels, fragmented or absent elastic fibers, and interstitial edema, all of which were present in our patient.

From a clinical perspective, the bullous variant represents a diagnostic pitfall. Its soft, translucent, cyst-like surface may mimic several unrelated conditions. Cystic hygroma, for instance, shares transillumination and a fluctuant consistency, but usually presents in infancy and lacks the firm, deeper component characteristic of pilomatricoma [11]. Hemangiomas or venous malformations may also resemble bullous pilomatricoma, yet Doppler flow studies typically reveal vascular channels, a feature absent in our patient’s ultrasonography [2]. Epidermal inclusion cysts and branchial cleft cysts may appear fluctuant and lobulated, but do not demonstrate the biphasic consistency or calcific foci commonly found in pilomatricoma. The preoperative diagnostic accuracy for pilomatricoma with or without bullous change remains low largely because its morphology overlaps with more common lesions, and because classic pilomatricoma features may not always be visually apparent [5].

Dermoscopy can be supportive but is not definitive. Features such as white areas corresponding to calcification, bluish areas correlating with basaloid cell proliferation, and linear, irregular vessels indicate tumor-induced vascular remodeling, but these findings may be inconsistent or obscured in bullous subtypes [5]. In our case, dermoscopy was inconclusive, illustrating the limits of this modality in diagnosing variants of pilomatricoma.

Ultrasonography may provide useful preoperative clues. Pilomatricoma often appears as a well-demarcated, mixed echogenicity mass with internal calcific foci and a hypoechoic rim reflecting inflammation or edema [2]. In bullous pilomatricoma, the superficial lymphangiectatic component may create a heterogeneous, sometimes multilayered appearance. The absence of Doppler flow in our patient further supported a non-vascular lesion, helping to narrow the differential diagnosis away from vascular malformations or hemangiomas. Nevertheless, imaging cannot definitively distinguish pilomatricoma from other benign adnexal or cystic lesions, reinforcing the necessity of histopathologic confirmation.

Fine-needle aspiration cytology (FNAC), though widely used for superficial lesions, is notoriously unreliable in pilomatricoma. Khan et al. describe high rates of misdiagnosis, largely due to sampling bias; aspirates may contain predominantly basaloid cells, mimicking malignancy, or ghost cells alone, mimicking epidermal cysts [6]. This reinforces that FNAC should be interpreted with caution in cases where pilomatricoma is suspected clinically.

The most remarkable aspect of this case lies in the presence of pilomatricoma debris within adjacent lymph node tissue. Several mechanisms might explain this phenomenon. One possibility is microscopic rupture of the tumor capsule, allowing keratin fragments to drain passively through dilated lymphatic channels into regional lymphoid tissue. Another plausible explanation is that the tumor was anatomically contiguous with a small lymph node, permitting direct extension of extruded debris without malignant infiltration. The extensive lymphangiectasia observed histologically provides a physiologic conduit through which keratinous material might migrate.

Importantly, lymph node involvement in this setting does not imply malignant transformation. Pilomatrix carcinoma, the malignant counterpart of pilomatricoma, displays marked cytologic atypia, infiltrative edges, high mitotic rates, necrosis, and aggressive local behavior; none of which were present in our patient [10]. Instead, the granulomatous reaction within the lymph node appears to represent a foreign-body response to keratin rather than true metastatic dissemination. Awareness of this possibility is essential for pathologists, as the presence of squamoid or keratin debris within lymph nodes can raise unwarranted suspicion for metastatic squamous cell carcinoma or adnexal carcinoma, potentially leading to overtreatment or unnecessary anxiety.

Therapeutically, pilomatricoma is managed by complete surgical excision, which is curative in nearly all cases. Recurrence rates are extremely low and generally attributable to incomplete excision [1]. The excellent postoperative outcome in our patient aligns with expectations in the literature, and the absence of recurrence at six months reassures the benign nature of the tumor despite its unusual histological behavior.

Overall, this case underscores several important teaching points. First, clinicians should consider bullous pilomatricoma in the differential diagnosis of translucent or cyst-like lesions that exhibit an underlying firm nodule. Second, imaging and dermoscopy, although helpful, may not reliably identify the bullous variant. Third, lymph node involvement by pilomatricoma debris, while rare, can occur and does not necessarily signify malignancy. Finally, early surgical excision provides definitive diagnosis, prevents complications related to rupture or inflammation, and ensures excellent prognosis. By documenting this unique presentation, we broaden the recognized spectrum of clinical and histopathologic features associated with bullous pilomatricoma and provide a framework for accurate diagnosis and management of similarly atypical cases.

## Conclusions

Bullous pilomatricoma is a rare variant of pilomatricoma that presents with a striking translucent, blister-like appearance over a firm tumor nodule. We reported a case of a 19-year-old male with a bullous pilomatricoma of the neck, notable for involvement of local lymphatic tissue, which produced a positive transillumination sign on exam. Histopathology revealed the characteristic features of pilomatricoma (basaloid cells, ghost cells, calcifications, and foreign-body giant cell reaction) along with marked dermal lymphatic dilatation and granulomatous inflammation extending into an adjacent lymph node. This case illustrates an unusual presentation that broadened the known behavior of pilomatricomas, yet the lesion remained a benign entity curable by complete excision. Clinicians should include pilomatricoma in the differential diagnosis of cystic-appearing skin nodules with mixed solid components, even in atypical locations or presentations. Awareness of the bullous variant can prevent misdiagnosis and ensure proper management. In summary, bullous pilomatricoma, though infrequent, is an important diagnostic consideration as it can mimic other translucid tumors; thorough clinical evaluation, imaging, and histological confirmation are essential. Early recognition and surgical treatment result in excellent outcomes with virtually no risk of recurrence.
